# Integration of Measurement-Based Care for Youth Depression and Suicidality Using VitalSign^6^

**DOI:** 10.1007/s10578-024-01680-8

**Published:** 2024-02-19

**Authors:** Tarrah B. Mitchell, Sarah M. Wakefield, Afsaneh Rezaeizadeh, Abu Minhajuddin, Ronny Pipes, Taryn L. Mayes, Joshua S. Elmore, Madhukar H. Trivedi

**Affiliations:** 1 Department of Psychiatry, Texas Tech University Health Sciences Center, Lubbock, TX USA; 2 Center for Depression Research and Clinical Care, Peter O’Donnell Jr. Brain Institute, Department of Psychiatry, University of Texas Southwestern Medical Center, Dallas, TX USA; 3 Peter O’Donnell Jr. School of Public Health, University of Texas Southwestern Medical Center, Dallas, TX USA

**Keywords:** Measurement-based care, Child, Adolescent, Depression, Suicide

## Abstract

Depression and suicidality are prevalent in youth and are associated with a range of negative outcomes. The current study aimed to evaluate a measurement-based care (MBC) software (VitalSign^6^) tool to improve the screening and treatment of depression and suicidality in youth aged 8–17 years within a rural, underserved population. To assess for depression and suicidality, the Patient Health Questionnaire-2 was administered as an initial screen, and the Patient Health Questionnaire-9 Modified for Adolescents (PHQ-9-A) was administered if the initial screen was positive. Data were collected at medical clinics over one year, and descriptive statistics and t-tests or Wilcoxon-Mann-Whitney tests were conducted. A total of 1,984 youth were initially screened (mean age of 13 years; 51.6% female); 24.2% screened positive for depression, and 14.9% endorsed suicidality. Of those who screened positive, the mean PHQ-9-A score was 12.8; 66.9% had PHQ-9-A scores in the moderate to severe range, and 44.2% endorsed suicidality. Almost half of the youth who screened positive for depression had at least one follow-up assessment, and about one quarter achieved remission 4 months after initial screening. Adolescents (12–17 years) had higher PHQ-9-A scores, higher suicidality, and more follow-up assessments than younger youth (8–11 years). Younger youth had higher rates of remission. The widespread use of MBC was feasible in this setting. It is important to utilize MBC to identify and treat youth with depression and suicidality and to do so in younger populations to improve their trajectory over time; VitalSign^6^ is one tool to help achieve these goals.

## Introduction

Depression, characterized by symptoms such as sadness, irritability, loss of interest or pleasure in activities, and hopelessness, among others, is one of the most common mental health diagnoses in youth and is associated with significant disability, morbidity, and mortality (CDC, 2023b). Further, suicidality, or the presence of suicidal thoughts or behaviors, is particularly common in youth with depression, and suicide is the second leading cause of death for youth between the ages of 10–14 and the third leading cause of death for youth between the ages of 15–17 (CDC, 2023a). Research has shown that the prevalence of depression has significantly increased between the years of 2005 and 2015 in the United States, with a more rapid rate of increase in youth compared to older individuals (Weinberger et al. 2018). Further, when compared to before the COVID-19 pandemic, the prevalence of depression in youth globally has doubled with estimates as high as 25.2%, and notable rates of increase in girls and older adolescents (Racine et al. 2021).

Because of the negative outcomes associated with depression in youth, the assessment, identification, diagnosis, and management of youth with depression is important, and guidelines and recommendations have been developed and endorsed by the U.S. Preventative Services Task Force and the American Academy of Pediatrics in line with this priority (e.g., Siu 2016; Zuckerbrot et al. 2018). These guidelines and recommendations highlight the importance of screening all youth over 12 years of age using evidence-based measures, providing accurate diagnoses, implementing/referring to evidence-based interventions, monitoring symptoms over time, and addressing safety concerns (Siu 2016; Zuckerbrot et al. 2018). Further, the American Academy of Child and Adolescent Psychiatry (2019) called for screening of youth ages 8 and older for depression in health care settings.

Measurement-based care (MBC) is an evidence-based practice of regularly assessing symptoms before or during a patient visit to inform clinical decision-making and treatment (Lewis et al. 2019). The MBC process includes administering validated measures, reviewing of the results by a provider, reviewing data with patients, and collaboratively evaluating and adjusting treatment plans based on the data (Jensen-Doss et al. 2020; Lewis et al. 2019). Several review articles, including those with youth, have shown that MBC leads to better patient outcomes compared to usual care (e.g., Shimokawa et al. 2010; Tam and Ronan 2017; Waldrop and McGuinness 2017).

Despite the support, MBC is underutilized (Lewis et al. 2019). There are several patient, provider, organization, and system barriers to implementation of MBC, including lack of knowledge or expertise, time, financial burden, concern about impacts on patient rapport, and lack of understanding of the benefits. Factors that increase the successful implementation of MBC include the use of brief, psychometrically sound measures, easy administration and scoring, integration with electronic health records, and available algorithms to support subsequent clinical decision-making (Lewis et al. 2019).

VitalSign^6^ is a software tool designed to facilitate the introduction of MBC into a variety of healthcare settings (Trivedi et al. 2019). This software was developed based on the idea that the management of depression is comparable to the management of other chronic illnesses, like diabetes, in that regular screening and assessment is useful for identifying patients in need of care and for tailoring treatment over time. VitalSign^6^ is available in English and Spanish, includes evidence-based measures, integrates with electronic health records, and eliminates several of the barriers to the implementation of MBC. The software allows quick and easy screening of patients, provides visual displays of data for provider use, and provides tailored treatment recommendations related to pharmacological, psychotherapeutic, and physical activity interventions (Trivedi et al. 2019).

Several studies have examined the integration of MBC for depression using VitalSign^6^. Notably, much of this work has been with adult populations from large metropolitan locations within primary care settings (e.g., Jha et al. 2019; Siniscalchi et al. 2020), though a few studies included samples of both adults and youth (> 12 years of age) within these settings (e.g., Kahalnik et al. 2019; Wang et al. 2022). Two studies were identified that examined the integration of VitalSign^6^ with child populations exclusively. Anton and colleagues (2021) utilized the software for MBC in a pediatric heart failure and transplant clinic, and Pop and colleagues (2019) utilized the software across clinic types in youth ages 10–17 years within a metropolitan location.

To fill gaps within the literature, the present study explores the implementation of VitalSign^6^ for MBC treatment of adolescent depression in a largely rural, medically underserved population. Additionally, this study explores the integration of VitalSign^6^ for the treatment of youth between the ages of 8 and 17, including younger patients than in previously identified studies. Identification and treatment earlier in development are important, as early intervention may result in less intervention needed over time and improved outcomes. This study makes an important contribution by examining follow-up data over time and exploring both depression and suicidality rates.

It was expected that the integration of VitalSign^6^ would allow for widespread screening of depression and suicidality for youth patients presenting to a range of clinic types within a rural, underserved population. Based on previous literature, it was hypothesized that rates of positive depression screenings would be between 17 and 25% (Pop et al. 2019; Racine et al. 2021) and rates of positive suicidality screenings would be between 7 and 18% (Anton et al. 2021; Bitsko et al. 2022). It was expected that regular follow-up assessments would be conducted in these clinics and that remission rates would be around 20% (Whiteford et al. 2013). Lastly, it was expected that depression and suicidality rates would be higher in adolescents (ages 12–17), compared to the younger youth (ages 8–10; Racine et al. 2021).

## Method

### Participants

The current study included youth patients between 8 and 17 years of age who presented to designated medical clinics between April 4, 2022 and March 31, 2023. For the larger VitalSign^6^ implementation, a total of nine clinics (one family medicine, one internal medicine, four pediatrics, one student health, and two psychiatry) at an academic health sciences center serving largely rural and underserved populations were included. These clinics were chosen as they allowed for routine follow-ups with patients, increasing the potential for continuous depression screening. The sample selected for the current paper includes only clinics that serve patients between the ages of 8 and 17 years (i.e., one family medicine, four pediatrics, and one psychiatry clinic). Most patients (98.5%) completed screenings in English. Staff members, such as nurses, front desk staff, and medical assistants helped assign and administer the assessments using VitalSign^6^. Clinicians that reviewed and integrated the assessment data into practice included licensed professional counselors, clinical social workers, psychologists, physicians, resident physicians, and advanced practice providers (e.g., nurse practitioners, physician associates).

### Procedures

The Institutional Review Board at the University of Texas Southwestern Medical Center (at which VitalSign^6^ was developed) reviewed the project and deemed it to be under the scope of a quality improvement project. The clinical administration and leadership teams of the designated clinics and the larger institution agreed to implement this MBC software as the standard of care. However, patients had the option of declining administration of the questionnaires at any time. Training was provided to clinic staff by the VitalSign^6^ implementation team. Self-paced virtual learning modules were made available to clinic staff and providers, which included a video about MBC and guided support in using VitalSign^6^ within the existing Electronic Health Records (EHR); quizzes were also interspersed to ensure understanding and knowledge before granting access to the software. The VitalSign^6^ implementation team also remained on site for approximately two months to aid in training, use, and support. The team was also available remotely to adjust workflows or provide technical support as needed. For more details about the VitalSign^6^ software, see the paper by Trivedi and colleagues (2019).

Each clinic was able to choose the frequency and order in which the MBC measures were presented to the patients. The suggestion included screening all patients (except for specimen or procedure visits) who were 8 years of age or older using the 2-item Patient Health Questionnaire (PHQ-2; Kroenke et al. 2001). If the initial depression screen was negative, depression would be reassessed in 12 months. If the patient screened positive for depression, the results would be reviewed in the EHR by a clinician, who would then collaborate with the patient on a recommended treatment plan. The diagnosis (as generated by a clinical interview) and recommended treatment plan could be saved in VitalSign^6^. If MBC was selected in VitalSign^6^, the patient would continue to receive depression measures at future treatment visits.

Measures could be administered during the clinic visit or as a pre-clinic visit activity. If a patient endorsed suicidality on the 9-item PHQ (PHQ-9) during a pre-clinic visit activity, an automatic message was displayed to the patient informing them that a clinician may not have immediate access to the information provided on the questionnaires. Steps for immediate assistance, crisis, or concerns related to depression or suicidality were listed. These steps included contacting a local or national mental health crisis line or calling 911. Clinicians were encouraged to quickly review the assessment data and respond accordingly.

### Measures

#### Patient Health Questionnaire (PHQ-2, PHQ-9, and PHQ-A)

All clinics assessed depression symptoms with a version of the PHQ (Kroenke et al. 2001). On the PHQ measures, the patients were asked to rate how often they have been bothered by each item on a scale from 0 (“Not at all”) to 3 (“Nearly every day”). The PHQ-2 (2 item version) was given as an initial screen; a score of 3 or more is considered a positive screen. If the PHQ-2 was positive in clinics serving patients between the ages of 8 and 17, the PHQ-A (PHQ 9-item Modified for Adolescents) was administered automatically (Johnson, Spitzer, and Williams, 2002). The PHQ-A was scored by totaling the responses to each of the nine items, with scores ranging from 0 to 27. Scores of 0–4 are considered minimal depression, scores of 5–9 are considered mild depression, scores of 10–14 are considered moderate depression, scores of 15–19 are considered moderately severe depression, and scores of 20–27 are considered severe depression. Additionally, item #9 on the PHQ-9-A asks about thoughts that the patient would be better off dead or of hurting themselves in some way; anything other than “Not at all” is considered a positive suicidality screen. The PHQ has been shown to have good psychometric properties (e.g., Gilbody et al. 2007; Kroenke and Spitzer 2002; Kroenke et al. 2001; Kroenke et al. 2010).

### Analytic Method

Descriptive statistics (e.g., means, medians, frequencies) were conducted using SAS statistical software (9.4, SAS Inc., Cary, NC). Younger children (ages 8–11) and adolescents (ages 12–17) were compared using t-tests or Wilcoxon-Mann-Whitney tests (as appropriate) in terms of continuous variables and using Chi-square tests in terms of categorical variables. Remission was defined by PHQ-9-A score < 5 at any follow-up visit after 4 months from the initial screening; this time interval was chosen in alignment with previous studies suggesting that 4 months (or about 18 weeks) is associated with lower likelihood of later relapse (Wang et al. 2022).

## Results

**Fig. 1:**
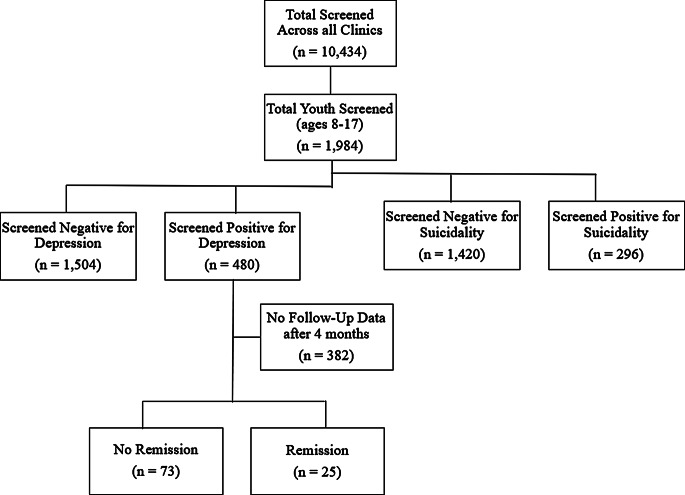
Flowchart of depression and suicidality screening in youth using VitalSign^6^

Across all clinics and ages, a total of 10,434 patients were screened during the year of data collection. A total of 1,984 patients who were initially screened were under the age of 18 years, with 553 between the ages of 8 and 11 years and 1,431 between 12 and 17 years. Of these youth patients, the mean age was 13.0 years, and 51.6% were female. Of these youth, 24.2% screened positive on the PHQ-2, and 14.9% endorsed suicidality. See Fig. 1 for more details.

Within the group of youth patients who screened positive on the PHQ-2, 64.4% were female, and the mean age was 13.2 years (see Table 1). The mean PHQ-2 score was 3.7, and the mean PHQ-9-A score was 12.8. Within this group, 33.1% had scores in the minimal/mild range, and 66.9% had scores in the moderate to severe range. Further, 44.2% of this group endorsed suicidality. Only about 38.5% of these patients had clinician data indicating the treatment selection. Of the data available, 14.4% of clinicians recommended the combination of pharmacological and nonpharmacological treatment, 9.6% recommended pharmacological treatment alone, 5.2% recommended nonpharmacological intervention alone, and the remaining 9.4% of clinicians selected to give an outside referral, to re-screen later, to have no follow-up, or that the family refused treatment. 40.8% of patients had at least one follow-up assessment, with about one quarter of patients (25.6%) having two or more follow-up assessments. The median days until the first follow-up was 35 days (IQR = 21, 70.5). Regarding remission, 20.4% of youth patients who screened positive had follow-up data at least 4 months after the initial screening. Of those, 25.5% reached remission.

**Table 1:** Data for youth who screened positive for depression at the initial screening using VitalSign^6^

		< 12 years *n* = 124	12–17 years *n* = 356	Total Youth *n* = 480	p-value
Age	Mean (SD)	10.0 (1.1)	14.3 (1.7)	13.2 (2.4)	< 0.0001
Sex	Female (n, %)	70 (56.4)	239 (67.1)	309 (64.4)	0.032
	Male (n, %))	54 (43.6)	117 (32.9)	171 (35.6)	
PHQ-2 Total	Mean (SD)	3.6 (0.9)	3.7 (1.0)	3.7 (0.9)	0.062
PHQ-9-A Total	Mean (SD)	10.9 (5.9)	13.5 (5.7)	12.8 (5.8)	< 0.0001
Suicidality	No (n, %)	85 (68.5)	183 (51.4)	268 (55.8)	0.0009
	Yes (n, %)	39 (31.5)	173 (48.6)	212 (44.2)	
PHQ-9-A Categories	Minimal (n, %)	14 (11.3)	23 (6.5)	37 (7.7)	0.0003
	Mild (n, %)	47 (37.9)	75 (21.1)	122 (25.4)	
	Moderate (n, %)	31 (25.0)	103 (28.9)	134 (27.9)	
	Moderately Severe (n, %)	18 (14.5)	99 (27.8)	117 (24.4)	
	Severe (n, %)	14 (11.3)	56 (15.7)	70 (14.6)	
Treatment Selection	Combination (n, %)	14 (11.3)	55 (15.4)	69 (14.4)	0.655
	Pharmacotherapy (n, %)	9 (7.3)	37 (10.4)	46 (9.6)	
	Non-Pharmacotherapy (n, %)	6 (4.8)	19 (5.3)	25 (5.2)	
	Outside Referral (n, %)	2 (1.6)	9 (2.5)	11 (2.3)	
	Treatment Refused (n, %)	0 (0.0)	2 (0.6)	2 (0.4)	
	Re-Screen (n, %)	4 (3.2)	14 (3.9)	18 (3.7)	
	No Follow-up (n, %)	5 (4.0)	9 (2.5)	14 (2.9)	
	Unknown/Missing (n, %)	84 (67.7)	211 (59.3)	295 (61.5)	
Number of Follow-up Visits*	0 (n, %)	89 (71.8)	195 (54.8)	284 (59.2)	0.011
	1 (n, %)	12 (9.7)	61 (17.1)	73 (15.2)	
	2 (n, %)	8 (6.4)	32 (9.0)	40 (8.3)	
	3+ (n, %)	15 (12.1)	68 (19.1)	83 (17.3)	
Days Until 1st Follow-up	Median (IQR)	40 (27, 61)	35 (20, 71)	35 (21, 70.5)	0.626
Remission Status**	No (n, %)	9 (52.9)	64 (79.0)	73 (74.5)	0.025
	Yes (n, %)	8 (47.1)	17 (21.0)	25 (25.5)	

*For *n* = 196 patients (*n* = 35 are < 12 years old, and *n* = 161 are 12–17 years old) with at least 1 follow-up visit after initial screening

**Remission status is calculated for 98 youth who had at least 4 months of follow-up after initial screening

When comparing age groups of youth who screened positive for depression, the mean age of the younger group (< 12 years) was 10 years, and the mean age of the older group (12–17 years) was 14.3 years (See Table 1). The older group had a higher percentage of females (*p* = 0.03). The mean PHQ-2 scores were not significantly different, but the mean PHQ-9-A score was significantly higher in the older group (*p* < 0.01); further, the symptom burden was higher in the older group, with greater percentages in the moderate to severe range (*p* < 0.01). Suicidality was significantly higher in the older group (*p* < 0.01). Treatment selection did not statistically differ by group. The number of follow-ups significantly differed by group, with the older group having more follow-up visits (*p* = 0.01); the days until the first follow-up visit did not significantly differ. Finally, remission differed by group, with the younger group having a higher percentage reaching remission status (*p* = 0.03).

## Discussion

The first hypothesis, that positive depression rates would be between 17 and 25% (Pop et al. 2019; Racine et al. 2021) and positive suicidality rates would be between 7 and 18% (Anton et al. 2021; Bitsko et al. 2022), was supported. The youth in the current sample reported rates at the higher end of the expected ranges, with 24% screening positive for depression and 15% screening positive for suicidality. These findings reveal that among youth presenting to medical clinics at an academic medical center serving largely rural and underserved populations, about one quarter of youth had significant depression symptoms, and many reported thinking they would be better off dead or of hurting themselves in some way. Further, findings indicate that the majority of those who screened positive for depression had moderate to severe symptoms, likely indicating a depressive or related disorder and a need for clinical intervention.

A second hypothesis, that regular follow-up assessments would be conducted in these clinics and that remission rates would be between around 20% (Whiteford et al. 2013), was also supported. Findings showed that a little less than half of the patients received at least one follow-up assessment after the initial screening and about one quarter received two or more follow-up assessments, indicating that clinicians assessed symptoms over time to inform treatment decisions. Further, in the subsample with available data for follow-up 4 months after the initial screening, about one quarter achieved remission. Therefore, MBC may be contributing to improvements in identification and treatment of youth with depression symptoms, which is promising; but given the lack of pre-implementation data in this manuscript this is impossible to verify. However, many more youth need ongoing support to achieve remission, and greater research is needed to aid in treatment algorithms to improve remission rates further.

The final hypothesis, that depression and suicidality rates would be higher in adolescents (ages 12–17) than younger youth (ages 8–10), was supported. Findings indicate that adolescents had higher PHQ-9-A scores and higher suicidality rates; these findings are consistent with previous literature (e.g., Racine et al. 2021). The younger youth were more likely to reach remission status, highlighting the importance of early identification and treatment of depression. This group started with less severe symptoms that were more likely to decrease and reach remission within 4 months. Use of MBC tools like VitalSign^6^ in youth under age 12 may improve the trajectory of symptom presentation in youth over time.

These findings must be interpreted considering limitations. First, additional demographic data of patients were not available. Information such as race/ethnicity and socioeconomic status of patients may be helpful to assess in future studies. Additionally, although the VitalSign^6^ software has the capacity to track data such as diagnoses and recommendations assigned by the treating clinicians, many of the clinicians in the current sample did not record this data in the software; 61.5% of patient treatment data is missing, for example. Missing data prevented many conclusions from being made about these variables. Further, data on actual treatment services received by youth (in addition to those services recommended by clinicians) are not available, limiting the granularity with which we can assess treatment decisions made by the providers. Work is currently underway to further integrate the VitalSign6 platform into the electronic health record, potentially allowing for the collection of more granular treatment data. Also, follow-up data at least 4 months from the initial screening was only available for 20% of the youth who screened positive for depression; this could be because the follow-up visit was sooner than 4 months, or the youth may not have presented for a follow-up visit yet within the current restricted time frame. Remission rates were reported on this subset of youth, which may not be an accurate representation of the larger population. Future studies should examine trajectories of depression and suicidality over longer time scales to further explore relapse and remission to identify more effective treatment strategies and algorithms. The Texas Youth Depression and Suicide Research Network (TX-YDSRN) is an example of this proposed work and is a state-wide research network focused on training clinicians in MBC to address youth depression and suicide, and on collecting and analyzing longitudinal data in treatment seeking youth (Trivedi et al., Under Review). The PHQ-A was originally designed for use in adolescents aged 12 and older (Johnson et al. 2002). Although recent work has used and validated the PHQ-A in children as young as 10 years of age (Horowitz et al. 2021; Tele et al. 2023), the measure has not been validated in children as young as age 8. As such, the results for patients under the age of 12 in this study should be interpreted cautiously. Future work will be needed to validate the measure in youth under age 12. Lastly, at the time of this study, qualitative acceptability data from patients who completed the assessments or clinicians who administered them were not available, though this information will be assessed and presented in future studies.

Despite these limitations, this study significantly contributes to the literature. First, it demonstrated that widespread use of MBC for depression and suicidality is feasible across a variety of clinics within an academic medical center serving largely rural and underserved populations. Additionally, this study included younger children (as young as 8 years of age) than previous studies using this software and compared data for younger youth and adolescents. Future studies with larger sample sizes should further explore differences in youth 8–11 years and 12–17 years.

## Summary

In conclusion, in this sample of youth between the ages of 8 and 17 years from academic medical center clinics serving rural and underserved populations, about 1 in 4 screened positive for depression and about 1 in 6 or 7 endorsed suicidality using a standardized screening process. Of those that screened positive for depression, and who had 4-month follow-up data, about a quarter reached remission, and remission rates were higher in those younger than 12 years of age compared to those older. It is important for clinics to utilize MBC to help identify and treat youth with depression and suicidality and to do so in younger populations to improve their trajectory over time; VitalSign^6^ is one software tool to help achieve these goals.
